# Neuroimaging mechanisms of acupuncture on functional reorganization for post-stroke motor improvement: a machine learning-based functional magnetic resonance imaging study

**DOI:** 10.3389/fnins.2023.1143239

**Published:** 2023-05-19

**Authors:** Mengxin Lu, Zhongming Du, Jiping Zhao, Lan Jiang, Ruoyi Liu, Muzhao Zhang, Tianjiao Xu, Jingpei Wei, Wei Wang, Lingling Xu, Haijiao Guo, Chen Chen, Xin Yu, Zhongjian Tan, Jiliang Fang, Yihuai Zou

**Affiliations:** ^1^Department of Neurology, Dongzhimen Hospital, Beijing University of Chinese Medicine, Beijing, China; ^2^Department of Acupuncture, Dongzhimen Hospital, Beijing University of Chinese Medicine, Beijing, China; ^3^Department of Chinese Medicine, Peking Union Medical College Hospital, Beijing, China; ^4^Department of Radiology, Dongzhimen Hospital, Beijing University of Chinese Medicine, Beijing, China; ^5^Department of Radiology, Guang’anmen Hospital, China Academy of Chinese Medical Sciences, Beijing, China

**Keywords:** stroke, motor recovery, minimal clinically important difference (MCID), acupuncture, machine learning, fMRI

## Abstract

**Objective:**

Motor recovery is crucial in stroke rehabilitation, and acupuncture can influence recovery. Neuroimaging and machine learning approaches provide new research directions to explore the brain functional reorganization and acupuncture mechanisms after stroke. We applied machine learning to predict the classification of the minimal clinically important differences (MCID) for motor improvement and identify the neuroimaging features, in order to explore brain functional reorganization and acupuncture mechanisms for motor recovery after stroke.

**Methods:**

In this study, 49 patients with unilateral motor pathway injury (basal ganglia and/or corona radiata) after ischemic stroke were included and evaluated the motor function by Fugl–Meyer Assessment scores (FMA) at baseline and at 2-week follow-up sessions. Patients were divided by the difference between the twice FMA scores into one group showing minimal clinically important difference (MCID group, *n* = 28) and the other group with no minimal clinically important difference (N-MCID, *n* = 21). Machine learning was performed by PRoNTo software to predict the classification of the patients and identify the feature brain regions of interest (ROIs). In addition, a matched group of healthy controls (HC, *n* = 26) was enrolled. Patients and HC underwent magnetic resonance imaging examination in the resting state and in the acupuncture state (acupuncture at the Yanglingquan point on one side) to compare the differences in brain functional connectivity (FC) and acupuncture effects.

**Results:**

Through machine learning, we obtained a balance accuracy rate of 75.51% and eight feature ROIs. Compared to HC, we found that the stroke patients with lower FC between these feature ROIs with other brain regions, while patients in the MCID group exhibited a wider range of lower FC. When acupuncture was applied to Yanglingquan (GB 34), the abnormal FC of patients was decreased, with different targets of effects in different groups.

**Conclusion:**

Feature ROIs identified by machine learning can predict the classification of stroke patients with different motor improvements, and the FC between these ROIs with other brain regions is decreased. Acupuncture can modulate the bilateral cerebral hemispheres to restore abnormal FC *via* different targets, thereby promoting motor recovery after stroke.

**Clinical trial registration:**

https://www.chictr.org.cn/showproj.html?proj=37359, ChiCTR1900022220.

## 1. Introduction

Stroke is the third-leading cause of death and disability worldwide, with ischemic stroke accounting for 62.4% (Collaborators, G. B. D. Stroke, 2021). Motor impairment is one of the main disabilities associated with stroke, causing a substantial social and psychological burden, and the concern about motor function recovery after stroke is growing (Stinear et al., 2020). The Fugl–Meyer Upper and Lower Extremity scales are recommended to be used as primary indicators of motor deficits and outcomes in stroke populations (Bushnell et al., 2015). A minimal clinically important difference (MCID) is the smallest improvement in an outcome measure that would be noticed as beneficial to a patient and be of clinical relevance, which is important to patient-centered care and evidence-based research (Embry and Piccirillo, 2020). The MCID may also be useful in advancing personalized medicine by characterizing those who are most likely to benefit from a treatment (Malec and Ketchum, 2020). Thus, the MCID for the Fugl–Meyer Assessment (FMA) is perceived as a meaningful recovery of motor function by post-stroke patients, deserving increasing interest and importance in medical practice and research.

Plasticity changes and functional reorganization occur spontaneously in post-stroke brains, and these alternations may contribute to the restoration of motor function following stroke (Murphy and Corbett, 2009; Wang et al., 2010; Dimyan and Cohen, 2011). Recent developments in functional magnetic resonance imaging (fMRI) have enabled the visualization of functional abnormalities and reorganization between brain regions or networks (Cramer et al., 2011). Degree centrality (DC) is a voxel-based analysis that can identify neural hubs associated with functional reorganization, reflecting the centrality or functional importance of the voxel or brain regions in whole-brain networks (Zuo et al., 2012; Zhang et al., 2017). One study found that DC was correlated with motor recovery after cerebral infarction (Liu et al., 2019). Moreover, functional connectivity (FC) is a seed-based analysis that demonstrates the temporal correlation across regions of interest (ROIs) (Carter et al., 2010) and is commonly used in neuroimaging studies. The joint application of DC and FC can better identify functional hubs associated with motor recovery and reveal local and global neurological remodeling after stroke. Both the recovery of motor function and neurological remodeling after stroke are crucial for the patient’s rehabilitation and may help to explore potential neural biomarkers. Clinical assessment of motor impairment combined with neuroimaging biomarkers of motor function can help to predict both motor recovery and motor outcomes, and stratify patients in clinical trials after stroke (Stinear, 2017). Thus, machine learning (ML) can help to implement and increasingly be used for diagnosis, prognosis prediction, and biomarkers selection for diseases (Zeng et al., 2018; Tu et al., 2021; Lian et al., 2022). Compared to *a priori* empirical or statistical comparisons, applying ML to select brain regions associated with motor recovery after stroke is more characteristic, personalized, and predictive.

Acupuncture is one of the traditional Chinese medical therapies that provides a positive effect in improving post-stroke symptoms and stroke rehabilitation (Wu et al., 2010). Studies have shown that acupuncture can improve motor dysfunction after stroke (Birch and Robinson, 2022) and has a certain safety profile (Zhang et al., 2005). However, the mechanisms of acupuncture remain elusive. Neuroimaging may be able to provide some evidence for the central nervous system effects of acupuncture in the treatment of stroke (Qi et al., 2014; Wu et al., 2016). Our previous studies have shown that acupuncture was able to modulate the disrupted patterns of the whole-brain network following the subcortical ischemic stroke (Han et al., 2020). Acupuncture triggered unique responses in the sensorimotor cortex in post-stroke hemiplegia patients, related to the neurological functional damage and the stage of stroke (Wang et al., 2022). Nowadays, ML studies of neuroimaging biomarkers exploration are increasing, especially in stroke recovery and acupuncture.

In the present study, we included stroke patients with impaired unilateral motor pathways, grouped them according to their MCID of the FMA over 2 weeks, and analyzed the DC of the whole brain. The L1-multi kernel learning machine (L1-MKL) in PRoNTo was used to select feature brain regions that could distinguish between these two groups of patients. Our primary aim of the present work was to search for differential ROIs between stroke patients with different manifestations of motor function recovery. Then we analyzed the FC between these selected ROIs and the whole brain, comparing abnormal functional connectivity between patients and healthy controls in the resting and acupuncture states. We hypothesized that (1) functional hubs with prediction-related features exist between patients with different motor recovery profiles, (2) these feature brain regions have different responses to the whole brain after stroke, and (3) acupuncture has its unique neuroimaging mechanisms.

## 2. Materials and methods

### 2.1. Participants

**Figure 1:**
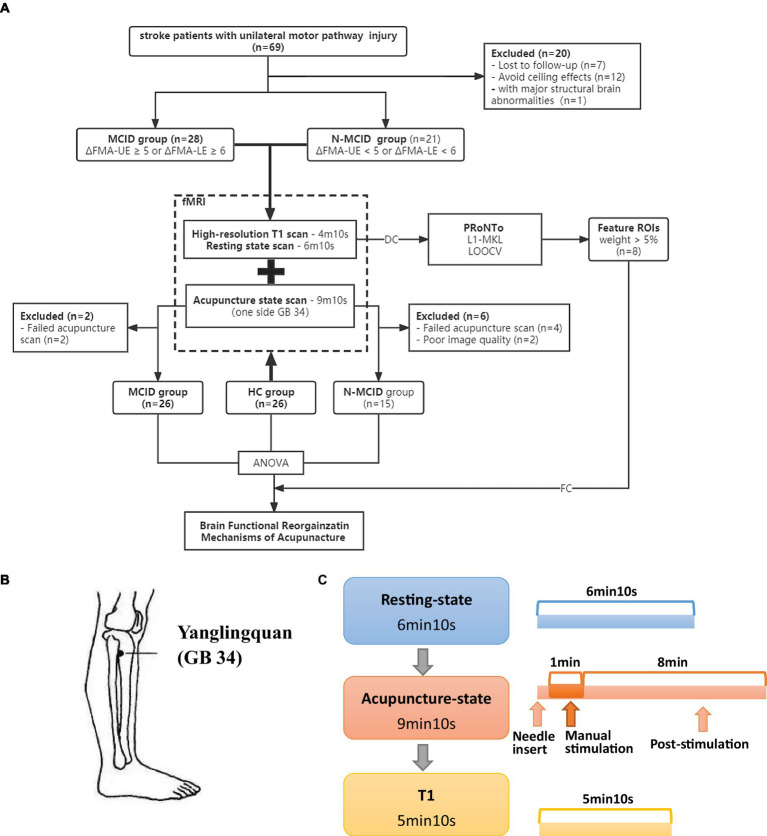
Study design. **(A)** The flowchart of the study protocol. **(B)** The location of Yanglingquan (GB 34). **(C)** The scanning protocol. FMA-UE, Fugl–Meyer assessment upper extremity scores; FMA-LE, Fugl–Meyer assessment lower extremity scores; HC, healthy controls; MCID, minimal clinically important difference; N-MCID, non-minimal clinically important difference; DC, degree centrality; FC, functional connectivity; ROI, regions of interest; L1-MKL, L1-multi kernel learning machine; LOOCV, leave-one-out cross-validation.

The present study included 69 stroke hemiplegia patients due to unilateral motor pathway (basal ganglia and/or corona radiata) injury. Patients were evaluated for motor function on the day of enrolment and 2 weeks later, and grouped according to the changes in motor function. In addition, a total of 26 healthy subjects were recruited as healthy controls (HC). Participants were enrolled at Dongzhimen Hospital Affiliated to Beijing University of Chinese Medicine and received clinical routine treatment during the follow-up period. All participants underwent MRI scans on the day of enrollment. The study was approved by Dongzhimen Hospital Affiliated to Beijing University of Chinese Medicine Institutional Review Boards (NO: DZMEC-KY-2018-58). All participants provided written informed consent. Figure 1A shows the study protocol.

The included patients met the following criteria: (1) patients with cerebral infarction whose course of the disease is 3 months and conform to the diagnostic criteria of cerebral infarction; (2) right-handed patients; (3) between 40 and 80 years of age, both men and women are eligible; (4) patients in whom the infarct was located in the unilateral basal ganglia and/or the radiation coronal region; (5) the patient with unconscious disorder and the condition is relatively stable; (6) The patient himself or his immediate family members sign the informed consent.

The exclusion criteria of patients were as following: (1) the patient had ever taken psychotropic drugs in the past months; (2) the patient being pregnant or lactation; (3) the patient had a history of neurologic or psychiatric disorders; (4) the patient had any other health problems or poor physical conditions that may influence participation; (5) the patient had any other brain structure damage or abnormalities identified by MRI examinations; (6) the patient had any history of alcohol or drug dependency; (7) the patient had any MRI contraindications.

The included healthy controls met the following criteria: (1) right-handed people; (2) between 40 and 80 years of age, both men and women are eligible; (3) people proved to be healthy by a medical examination; (4) people signed the informed consent and volunteered to participate in the experiment.

The exclusion criteria of healthy controls were as following: (1) the person had ever taken psychotropic drugs in the past months or had a family genetic history of the mental and nervous system; (2) the person being pregnant or lactation; (3) the person had a history of neurologic or psychiatric disorders; (4) the person had any other health problems or poor physical conditions that may influence participation; (5) the person had any other brain structure damage or abnormalities identified by MRI examinations; (6) the person had any history of alcohol or drug dependency; (7) the person had any MRI contraindications; (8) the person was conducted to other similar research.

### 2.2. Clinical evaluation and grouping

We evaluated the motor impairment of patients by the Fugl–Meyer Assessment (FMA). FMA includes assessments of the upper extremity (FMA-UE) and lower extremity (FMA-LE) (Sullivan et al., 2011). Two professional neurologists evaluated their motor function at baseline and 2-week follow-up. We grouped patients according to the changes of FMA scores (∆FMA = FMA_follow-up_ - FMA_baseline_). We divided patients with an ∆FMA-UE ≥ 5 or ∆FMA-LE ≥ 6 (Bushnell et al., 2015; Pandian et al., 2016) into the MCID group (*n* = 28, 17 male), and those with an ∆FMA-UE < 5 or ∆FMA-LE < 6 (Page et al., 2012) into the N-MCID (Non-MCID) group (*n* = 21, 15 male). To avoid the ceiling effects, patients with less severe motor impairment (FMA-UE > 61 or FMA-LE > 28) were not included in the analysis (Gladstone et al., 2002).

### 2.3. Image data acquisition

MRI data were acquired using a 3.0 Tesla Siemens scanner (MAGNETOM Verio Siemens Medical Systems, Erlangen, Germany) with a 32-channel head coil. For resting-state and acupuncture-state fMRI scans, participants were instructed to keep their eyes closed and stay awake without performing any cognitive tasks. The imaging parameters of the EPI sequence were as follows: repetition time (TR) = 2000 ms, echo time (TE) = 30 ms, slice number = 31, thickness = 3.5 mm, flip angle = 90°, and matrix size = 64 × 64. High-resolution structural images (T1) were acquired through a magnetization-prepared rapid acquisition with gradient-echo (MPRAGE) sequence with the following parameters: TR/TE = 1900/2.53 ms, field of view (FOV) = 250 × 250 mm^2^, matrix size = 256 × 256, flip angle = 9°, slice number = 176, and slice thickness = 1 mm.

### 2.4. Details of acupuncture operations

The acupuncture operations were on the affected side in patients, or the left side in HC during the acupuncture state fMRI scans. The needles were disposable sterile silver needles (specification parameter: φ0.40 × 40 mm, purchased from Beijing Zhongyantaihe Medical Instrument Co., LTD., manufactured by Suzhou Shenlong Medical Instrument Co., LTD). Yanglingquan (GB 34) is located on the outside of the lower leg, in the middle of the concavity of the anterior and inferior parts of the fibula head. Acupoint selection is performed according to the National standard GB/T 12346–2006 Name and Location of Acupoints. The position of GB 34 is shown in Figure 1B. After the routine skin disinfection, the needle was vertically inserted for 1–1.5 Cun (about 15–25 mm depending on the height and weight of a participant) at GB 34. There was a 10 s post-onset phase of the resting state with the needle inserted, followed by a 1-min manual stimulation phase by using the mild reinforcing-reducing method at the frequency of 1 Hz. Then, an 8-min post-stimulation phase occurred with the needle remaining inside the leg. The needle was removed and disposed of after the acupuncture scanning.

### 2.5. Preprocessing of fMRI data

The structural and functional MRI images were preprocessed using Data Processing & Analysis for Brain Imaging (DPABI^1^) (Yan et al., 2016), which is based on Statistical Parametric Mapping (SPM 12^2^). These toolboxes were based on Matlab 2017a (Mathworks, Sherborn, MA).

All images were flipped to constrain the lesion’s location to the right brain hemisphere (i.e., all patients’ lesions were constrained to be on the positive MNI x-coordinates by simply inverting the signal of the voxels along the x-axis when the lesion was located on the left hemisphere).

#### 2.5.1. Preprocessing of resting state fMRI data

The first 10 volumes of each participant were discarded as usual. Slice timing and head motion correction were conducted for the remaining time points. Participant data were excluded if they met the head motion criteria, which included head motion >3 mm translation or a 3° rotation in any direction. In order to achieve better registration, functional and anatomical images were manually reoriented to the anterior commissure. A linear transformation was used to co-register anatomical images to the functional images for each subject. Subsequently, the transformed anatomical images were segmented into gray matter, white matter, and cerebrospinal fluid by using the new segmentation tool in SPM 12 (Ashburner and Friston, 2005). The transformation from individual space to Montreal Neurological Institute (MNI) space was computed and resampled at a resolution of 3 mm × 3 mm × 3 mm voxels. Next, a Friston 24-parameter model was used to regress out the effects of head motion (Friston et al., 1996). Other nuisance variables, including white matter signal, cerebrospinal fluid signal, and global signal were regressed out from the time series of all voxels *via* linear regression. Then, the images were smoothed using a 6 mm full-width-at-half-maximum Gaussian kernel. After, a temporal filter (0.01–0.08 Hz) was applied to reduce physiological noise at other frequency bands. Finally, we manually checked each subject’s structural and functional images to promise the quality of data.

#### 2.5.2. Preprocessing of acupuncture state fMRI data

The first 45 volumes of each participant were discarded (during this period of time, we inserted the needle and stimulated). The other steps of preprocessing were the same to resting state fMRI data, and we also checked the data manually.

### 2.6. Degree centrality measurement

Weighted DC measures were calculated using DPABI. To obtain each subject’s graph, we computed the Pearson correlation coefficients between any pairs of voxels. Each voxel acted as a node in the graph, and each significant Pearson correlation between any pair of voxels represented an edge. An *n* × *n* matrix of Pearson correlation coefficients between any pair of voxels was obtained for each subject by thresholding each correlation at *r* > 0.2 to eliminate possible spurious connectivity. Then, the weighted DC strength of a voxel was computed as the sum of the connectivity between a given brain voxel and all other voxels. Finally, the individual-level voxel-wise DC value for each subject was converted into a *Z*-score map by the Fisher-*Z* transformation to improve normality.

### 2.7. PRoNTo analyses

To classify two groups, we used L1-MKL from the PRoNTo toolbox^3^ (Schrouff et al., 2013). Classification based on L1-MKL is viewed as a supervised learning algorithm because it facilitates learning a model from training data whose class label was previously defined and assigns class labels to test data. Support Vector Machines (SVM) transformed low dimensional data into a higher dimension and generated support vector classifiers that separated higher dimensional data into two groups *via* kernel functions (Lanckriet et al., 2004; You et al., 2021).

#### 2.7.1. Regions of interest-based machine kernel learning

Degree centrality maps were served as inputs to classify two groups in machine learning. The ROIs are defined on the basis of Craddock’s work, which generated an ROI atlas by parcellating whole brain resting-state fMRI data into spatially coherent regions of homogeneous FC (Craddock et al., 2012). For each participant, 200 features were extracted from 200 ROIs as an machine kernel learning (MKL) source (ROI-MKL). Features were selected to form a kernel matrix through a multi-kernel strategy. A nested cross-validation (CV) scheme was used to obtain unbiased estimates of classification performance.

#### 2.7.2. Performance evaluation of classification methods

Machine learning applications apply a leave-one-out cross-validation (LOOCV) strategy with an optimized nested hyper-parameter meter range of 2.^[−5:5] to evaluate the generalizability of classifiers (Wen et al., 2017). Overall classification accuracy, sensitivity (i.e., the proportion of MCID patients correctly classified), and specificity (i.e., the proportion of N-MCID patients correctly classified) can be defined from CV results quantifying the performance of classifiers. *Value of ps* were calculated using permutation tests (1,000 permutations).

#### 2.7.3. Weights map

Next, anatomical atlas weights were computed to visualize the relative importance of each region in the multivariate pattern analysis decision function displaying regional patterns of the DC maps. The weight of each feature in ROIs can also be obtained because the coefficient is learned as a single optimization problem in equations and weights relevant to each kernel. In this study, each kernel was known as an “ROI-weight” that reflected “voxel-weight.” Higher absolute indicator weight values discriminated corresponding features. Because there is no conventional threshold for the optimal number of ROIs to be retained, in this exploratory study we presented the ROIs that weight vector value more than 5%, called feature ROIs, number of eight.

### 2.8. Functional connectivity measurement

The voxel-wise functional connectivity analyses between each ROI (the feature ROIs) and each voxel in the brain areas were performed to generate seed-based FC maps at baseline and at 2 weeks after stroke. For group analyses, the correlation coefficients were transformed to *Z* values using Fisher’s *Z*-transformation to improve the normality of the correlation coefficient.

### 2.9. Statistical analysis

All data were analyzed using the statistical program SPSS 25.0 for intergroup comparisons of demographic data and FMA scores, and the Shapiro–Wilk test was used to verify the normality of the data. Subject characteristics were compared among three groups using ANOVA or the Mann–Whitney *U* test depending on their distributions, and two groups using a two-sample *t*-test or the Mann–Whitney U test depending on their distributions. The proportions of sex and lesion side were examined using the chi-square test. *p* < 0.05 indicated statistical significance.

The statistical analysis was conducted by DPABI software. The mean framewise displacement (i.e., Mean FD_Jenkinson) was taken as the covariate to control the impact of unnecessary head motion in the statistical analysis. One-way ANOVA (*p* < 0.05, Bonferroni corrected) in the statistical analysis module of DPABI software was performed to compare variables among the three groups (HC, MCID, and N-MCID). A mask was built according to the results of ANOVA. Based on the mask, the inter-group differences were obtained by using the *post hoc t*-test. Two-tailed Gaussian random field (GRF) correction (voxel threshold of *p* < 0.01 and cluster threshold of *p* < 0.05) was performed during the two-sample *t*-test (the *post hoc* test).

## 3. Results

### 3.1. Demographic and clinical data

**Figure 2:**
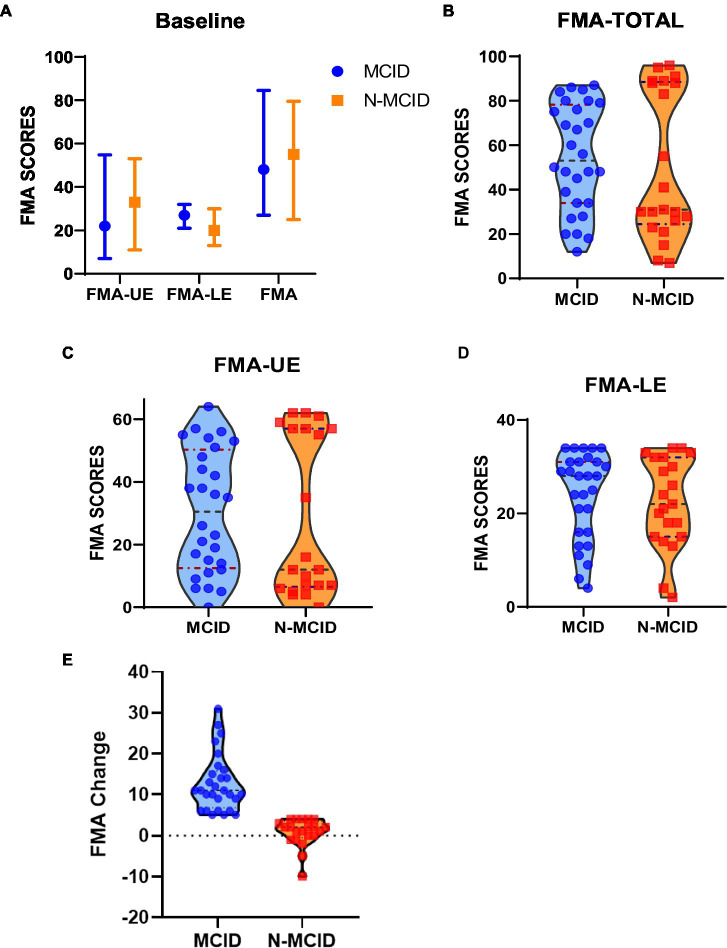
Fugl–Meyer assessment (FMA) scores at baseline for each group of stroke patients. **(A)** The median, upper and lower quartile distribution of FMA at baseline for each group. **(B)** The distribution of FMA-TOTAL for the two groups. **(C)** The distribution of FMA-UE for the two groups. **(D)** The distribution of FMA-LE for the two groups. **(E)** The distribution of FMA-Change for the two groups. The blue dots represent patients in the MCID group; the orange squares represent patients in the N-MCID group. FMA-UE, Fugl–Meyer assessment upper extremity scores; FMA-LE, Fugl–Meyer assessment lower extremity scores; patients. FMA-TOTAL, Fugl–Meyer assessment total extremity scores; MCID, minimal clinically important difference; N-MCID, non-minimal clinically important difference.

A total of 69 patients were registered for this study period. After excluding 7 patients with unavailable 2-week FMA scores, 12 patients with ceiling effects, and 1 patient with major structural brain abnormalities, 49 patients were finally included. The drop-out rate was 10.14%. The included patients were divided into the MCID group (*n* = 28, 17 male) and the N-MCID group (*n* = 21, 15 male). There was no significant difference between the MCID and N-MCID in sex (χ^2^ = 0.608, *p* = 0.436), age (*Z* = −0.101, *p* = 0.919), lesion side (*χ*^2^ = 1.612, *p* = 0.204), course of disease (*Z* = −0.192, *p* = 0.848), and FMA scores at baseline. Table 1 and Supplementary Table S1 show the demographic and clinical information of both groups of patients. Figure 2 shows the distributions of motor function between the two groups of patients at baseline.

**Table 1:** Demographic and clinical data.

Characteristics	Group			
	MCID (*n* = 28)	N-MCID (*n* = 21)	χ^2^/Z	Value of *p*
Sex (male/female)	17/11	15/6	0.608	0.436^a^
Age (years)	62.00(57.00–67.75)	62.00(53.50–69.00)	−0.101	0.919^b^
Lesion side (left/right)	13/15	6/15	1.612	0.204^a^
Course of disease (days)	18.50(9.50–30.75)	21.00(6.50–31.00)	−0.192	0.848^b^
FMA-UE	22.00(7.00–54.75)	33.00(11.00–53.50)	−0.354	0.723^b^
FMA-LE	27.00(21.00–32.00)	20.00(13.00–30.00)	−1.600	0.110^b^
FMA-total	48.00(27.00–84.50)	55.00(25.00–79.50)	−0.455	0.649^b^

The data are presented as the median (interquartile range) for non-normally distributed data. ^a^The value of *p* was obtained by a chi-square test; ^b^The value of *p* was obtained by a two-sample nonparametric test. MCID, minimal clinically important difference; N-MCID, non-minimal clinically important difference; FMA-UE, Fugl–Meyer assessment upper extremity scores; FMA-LE, Fugl–Meyer assessment lower extremity scores; FMA-Total, Fugl–Meyer assessment total scores.

In the acupuncture state scanning, 7 patients failed to complete this fMRI state scanning and 2 patients were excluded for poor image quality. Finally, 26 patients in the MCID group, 15 patients in the N-MCID group, and 26 age (χ^2^ = 0.131, *p* = 0.937) and sex (*χ*^2^ = 1.363, *p* = 0.506) matched healthy controls (16 male) were included. Table 2 shows the demographic and clinical information of all the participants who completed the acupuncture-state scanning.

**Table 2:** Demographic and clinical data of acupuncture state.

Characteristics	Group				
	MCID (*n* = 26)	N-MCID (*n* = 15)	HC (*n* = 26)	χ^2^/Z	Value of *p*
Sex (male/female)	16/10	10/5	16/10	0.131	0.937^a^
Age (years)	62.00(56.50–66.25)	61.00(50.00–66.00)	59.50(53.75–62.25)	1.363	0.506^b^
Lesion side (left/right)	13/13	4/11		2.134	0.195^a^
Course of disease (days)	20.50(12.50–35.00)	28.00(18.00–42.00)		−1.300	0.194^b^
Motor assessment					
FMA-UE	35.50(13.50–51.25)	12.00(5.00–55.00)		−1.070	0.285^b^
FMA-LE	28.00(17.00–31.00)	21.00(15.00–32.00)		−0.976	0.329^b^
FMA-Total	54.00(34.00–79.00)	30.00(21.00–87.00)		−0.921	0.357^b^

The data are presented as the median (interquartile range) for non-normally distributed data. ^a^The value of *p* was obtained by a chi-square test; ^b^The value of *p* was obtained by a nonparametric test. MCID, minimal clinically important difference; N-MCID, non-minimal clinically important difference; HC, healthy controls; FMA-UE, Fugl–Meyer assessment upper extremity scores; FMA-LE, Fugl–Meyer assessment lower extremity scores; FMA-Total, Fugl–Meyer assessment total scores.

### 3.2. Results of feature ROIs identified by machine learning classification

**Figure 3:**
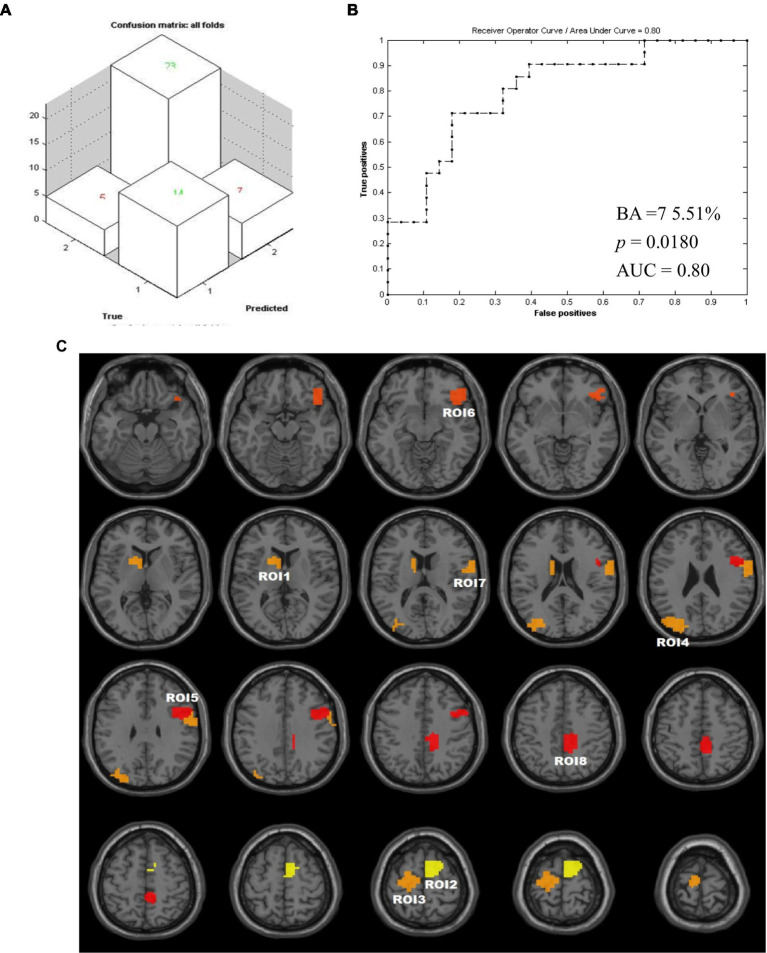
Results of machine learning. **(A)** The confusion matrix output by this classification. (1) i.e., N-MCID; (2) i.e., MCID. **(B)** Receiver operating characteristic curve and area under curve for the classification developed with two groups as inputs. The value of *p* was obtained during 1,000 permutation testing. **(C)** Weights map of all feature ROIs. The color of the clusters from red to yellow represents that the weight becomes larger. AUC, area under curve; BA, balanced accuracy; ROI, regions of interest.

The ML analysis was able to classify the MCID and N-MCID groups with 75.51% balanced accuracy (BA, *p* = 0.018 during 1,000 permutation testing), based on DC of whole brain functional regions maps. Specifically, class accuracy was 82.14% (23/28) for the MCID group and 66.67% (14/21) for the N-MCID group. In addition, the class predictive value was 76.67% for the MCID group and 73.68% for the N-MCID group. The AUC was 0.800. Figures 3A,B illustrate the performance of machine learning classification. The PRoNTo identified several functional regions with weights used by the decision function of the machine to predict group classification. Figure 3C shows the weight maps of all the regions with prediction weights.

We remained 8 feature ROIs with relatively high predictive weights for the group classification, including the bilateral supplementary motor areas (SMA), precentral gyrus (PreCG), postcentral gyrus (PoCG), paracentral lobule (PCL), etc.; the contralesional caudate nucleus (CAU), putamen (PUT), middle occipital gyrus (MOG), superior occipital gyrus (SOG), angular gyrus (ANG), etc.; and the ipsilesional dorsolateral superior frontal gyrus (SFG), inferior frontal gyrus (IFG), orbital gyrus, middle cingulate and paracingulate gyri (MCC), precuneus (PCUN), etc. Table 3 lists the regions with weights >5% and the intergroup comparisons of DC. Moreover, we compared DCs of these feature ROIs between groups. Compared to N-MCID, MCID exhibited significantly higher DC in ROI 1 (i.e., the left caudate nucleus and the left putamen, *t* = −2.016, *p* = 0.049) and ROI 2 (i.e., the right SMA and the right SFG, *t* = −3.042, *p* = 0.004), indicating that MCID had stronger nodal centralities in these brain regions. However, there were no significant differences between the other ROIs, indicating that group comparisons of DC cannot classify two groups.

**Table 3:** Weights of feature ROIs and comparison of DC.

Feature ROIs	ROI weight (%)	Brain regions in AAL	Hemi-sphere	DC	
				*t*	Value of *p*
ROI 1	17.67	Caudate nucleus (CAU)	L	−2.016	0.049
		Lenticular nucleus, putamen (PUT)			
ROI 2	12.02	Supplementary motor area (SMA)	R	−3.042	0.004
		Superior frontal gyrus, dorsolateral (SFG)			
ROI 3	8.86	Precentral gyrus (PreCG)	L	1.972	0.054
		Paracentral lobule (PCL)			
		Supplementary motor area (SMA)			
		Superior frontal gyrus, dorsolateral (SFG)			
		Postcentral gyrus (PoCG)			
ROI 4	8.63	Middle occipital gyrus (MOG)	L	2.337	0.024
		Superior occipital gyrus (SOG)			
		Angular gyrus (ANG)			
ROI 5	8.42	Inferior frontal gyrus, opercular part (IFGoperc)	R	0.742	0.462
		Precentral gyrus (PreCG)			
		Middle frontal gyrus (MFG)			
		Inferior frontal gyrus, triangular part (IFGtriang)			
ROI 6	7.53	Inferior frontal gyrus, orbital part (IFGorb)	R	1.823	0.075
		Posterior orbital gyrus (OFCpost)			
		Lateral orbital gyrus (OFClat)			
		Insula (INS)			
		Temporal pole: superior temporal gyrus (TPOsup)			
		Inferior frontal gyrus, triangular part (IFGtriang)			
		Anterior orbital gyrus (OFCant)			
ROI 7	6.75	Postcentral gyrus (PoCG)	R	0.575	0.568
		Precentral gyrus (PreCG)			
		Rolandic operculum (ROL)			
		Inferior frontal gyrus, opercular part (IFGoperc)			
ROI 8	6.03	Middle cingulate and paracingulate gyri (MCC)	R	−1.228	0.226
		Precuneus (PCUN)			
		Paracentral lobule (PCL)			
		Supplementary motor area (SMA)			

Regions were identified by setting the threshold to ≥5% of the maximum ROI-weight rank. Negative *t*-test values reflect significantly more DC in identified regions of interest for the MCID group. The value of *p* was based on two-sample *t*-tests. ROI, regions of interest; DC, degree centrality; L, left, i.e., contralesional hemisphere; R, right, i.e., ipsilesional hemisphere.

### 3.3. Functional connectivity and acupuncture effects of feature ROIs

**Figure 4:**
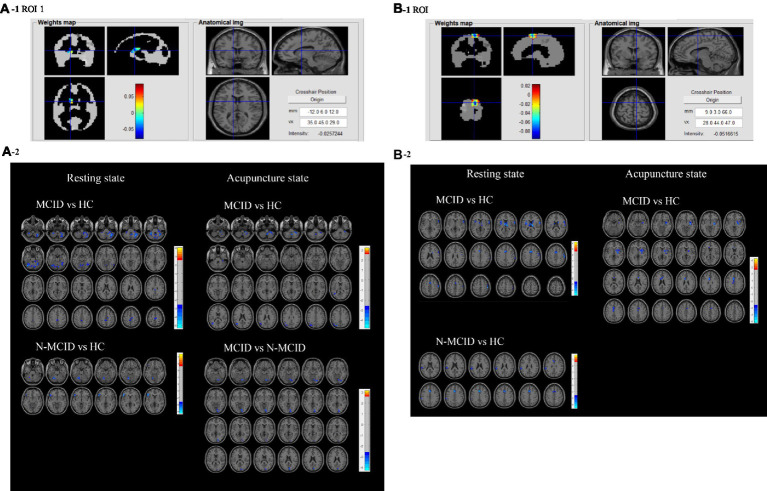
**(A1,B1)** Present the anatomical position and weights distribution of feature ROl 1 and ROl 2 Red indicates high weight and blue indicates low weight. **(A2,B2)** Plots of FC differences between feature ROl 1 and ROl 2 with the whole brain in the resting and acupuncture states. The blue clusters in the former group indicate lower FC between thee brain regions with the ROl, while the red clusters indicate higher FC between these brain regions with the ROl compared to the latter group. FC, functional connectivity; ROI, regions of interest; MCID, minimal clinically important difference; N-MCID, non-minimal clinically important difference. The FCs of the other ROIs are shown in Supplementary Figure S1.

Eight feature ROIs were used as seed-points to analyze the FC with the whole brain and the immediate effects of acupuncture among MCID, N-MCID, and HC. Table 4; Figure 4; Supplementary Figure S1 show the results of FC from ANOVA among three groups in the resting state and acupuncture state (*GRF* correction, voxel-*p* < 0.01, cluster-*p* < 0.05). In the resting state, the MCID group exhibited significantly lower FCs between almost all the ROIs (no significant difference in ROI 6) with other brain regions compared to HC (e.g., ROI 1 with the bilateral cerebellum, bilateral MCC, bilateral PCL, and right SMA), but higher FC only between ROI 8 (i.e., the right MCC, right precuneus, right PCL, and right SMA) with the left middle temporal gyrus (MTG) and left inferior temporal gyrus (ITG). Compared to HC, the N-MCID group similarly exhibited significantly lower FCs between the ROIs (i.e., ROI 1–3, ROI 5, and ROI 7–8) with other brain regions (e.g., ROI 2 with the bilateral anterior cingulate, left supramarginal gyrus, and left superior temporal gyrus), but higher FC between ROI 4 (i.e., the left MOG, left SOG, and left ANG) with the bilateral posterior cingulate gyrus (PCC). The FC between ROI 5 (i.e., the right IFG opercular part, right PreCG, right middle frontal gyrus, and right IFG triangular part) with the left cerebellar, left lingual gyrus (LING), left inferior occipital (IOG), and left MOG were lower in the MCID group than in the N-MCID group. There were no significant differences in FC between the other ROIs in the MCID group and the N-MCID group, but MCID revealed a wider range of lower FC, implying more generalized abnormal FC.

**Table 4:** Functional connectivity (FC) of feature ROIs in the resting state and acupuncture state.

Feature ROIs	Groups	Resting state			Acupuncture state		
		Clusters	Brain regions	*F*-value (peak)	Clusters	Brain regions	*F*-value (peak)
ROI 1	MCID vs. HC	1	Bilateral Cerebellum	−2.576	1	Left Middle occipital gyrus Middle temporal gyrus Angular gyrus	−2.577
		2	Bilateral Medial cingulate gyrus Paracentral lobule Right Supplementary motor area	−2.578	2	Bilateral Cerebellum	−2.579
	N-MCID vs. HC	1	Left Inferior frontal gyrus, triangular part Inferior frontal gyrus pars orbitalis	−2.586	NA		
		2	Left Cerebellum	−2.577			
	MCID vs. N-MCID	NA			1	Right Lingual gyrus Cerebellum Calcarine fissure and surrounding cortex Cuneate Inferior occipital gyrus	−2.576
ROI 2	MCID vs. HC	1	Bilateral Thalamus Caudate nucleus	−2.582	1	Right Pallidus Putamen Insula Hippocampus Amygdala Caudate nucleus	−2.576
		2	Bilateral Medial cingulate gyrus Supplementary motor area	−2.605	2	Bilateral Anterior cingulate and paracingulate gyri Medial cingulate and paracingulate gyri Right Supplementary motor area	−2.580
		3	Right Precentral gyrus Postcentral gyrus Supplementary motor area	−2.578			
		4	Right Rolandic operculum Inferior frontal gyrus, opercular part Temporal pole: Superior temporal gyrus Superior temporal gyrus	−2.589			
		5	Left Insula Rolandic operculum	−2.578				
	N-MCID vs. HC	1	Bilateral Anterior cingulate and paracingulate gyri Medial cingulate and paracingulate gyri	−2.593	NA		
		2	Left Supramarginal gyrus Middle temporal gyrus	−2.577			
	MCID vs. N-MCID	NA			NA		
ROI 3	MCID vs. HC	1	Right Superior parietal gyrus Postcentral gyrus Precuneus	−2.576	1	Right Precentral gyrus Postcentral gyrus	−2.579
		2	Right Precentral gyrus Middle frontal gyrus	−2.584	2	Left Middle occipital gyrus Inferior occipital gyrus	−2.578
		3	Right Caudate nucleus Thalamus	−2.594			
		4	Right Rolandic operculum Inferior frontal gyrus, opercular part	−2.580			
	N-MCID vs. HC	1	Right Caudate nucleus Thalamus	−2.583	NA		
	MCID vs. N-MCID	NA			NA		
ROI 4	MCID vs. HC	1	Right Middle occipital gyrus Superior occipital gyrus Precuneus	−2.578	1	Right Middle temporal gyrus Middle occipital gyrus Superior occipital gyrus	−2.578
		2	Right Precuneus Calcarine fissure and surrounding cortex Lingual gyrus	−2.586	2	Right Calcarine fissure and surrounding cortex Lingual gyrus	−2.577
		3	Right Superior parietal gyrus Precuneus posterior central gyrus	−2.580	3	Bilateral Precuneus	−2.577
	N-MCID vs. HC	1	Bilateral Posterior cingulate gyrus	4.595	NA		
	MCID vs. N-MCID	NA			1	Left Middle temporal gyrus Supramarginal gyrus Angular gyrus	−2.584
ROI 5	MCID vs. HC	NA			1	Left Inferior parietal gyrus Superior temporal gyrus Superior parietal gyrus Supramarginal gyrus Middle temporal gyrus Precuneus Posterior central gyrus	−2.577
					2	Bilateral Cingulate gyrus Left Supplementary motor area	−2.577
					3	Left Insula Inferior frontal gyrus, opercular part Precentral gyrus Rolandic operculum	−2.578
	N-MCID vs. HC	1	Right Caudate nucleus Thalamus	−2.581	1	Left Precuneus Cerebellum Posterior cingulate gyrus Bilateral Lingual gyrus	4.324
					2	Bilateral Middle frontal gyrus pars orbitalis Gyrus rectus	3.941
	MCID vs. N-MCID	1	Left Cerebellum Lingual gyrus Middle occipital gyrus Inferior occipital gyrus	−2.577	1	Bilateral Cerebellum	−2.576
					2	Left Middle temporal gyrus Inferior temporal gyrus Heschl’s gyrus	−2.577
ROI 6	MCID vs. HC	NA			1	Left Inferior frontal gyrus pars orbitalis Middle frontal gyrus Insula Inferior frontal gyrus, triangular part Temporal pole: superior temporal gyrus Anterior cingulate and paracingulate gyri Inferior frontal gyrus, opercular part Superior frontal gyrus, medial Superior frontal gyrus Superior temporal gyrus	−2.577		
					2	Bilateral Cerebellum	−2.578					
					3	Left Inferior temporal gyrus Middle temporal gyrus	−2.579
					4	Left Inferior parietal gyrus Angular gyrus Middle temporal gyrus supramarginal gyrus	−2.577
	N-MCID vs. HC	NA			NA		
	MCID vs. N-MCID	NA			1	Bilateral Cerebellum	−2.578
					2	Left Precuneus Superior parietal gyrus Inferior parietal gyrus Posterior central gyrus	−2.577
					3	Left Inferior temporal gyrus Middle temporal gyrus Inferior occipital gyrus	−2.576
ROI 7	MCID vs. HC	1	Bilateral Posterior central gyrus Precuneus Left Precentral gyrus Right Superior parietal gyrus	−2.578	1	Left Posterior central gyrus Precentral gyrus Paracentral lobule Supramarginal gyrus Inferior parietal gyrus Right Supplementary motor area Paracentral lobule	−2.576
		2	Left Middle temporal gyrus Superior temporal gyrus Insula	−2.576	2	Right Supramarginal gyrus Inferior parietal gyrus Angular gyrus	3.771
		3	Bilateral Supplementary motor area Left Superior frontal gyrus Paracentral lobule	−2.580	3	Left Middle temporal gyrus Superior temporal gyrus Middle occipital gyrus Inferior occipital gyrus Insula	−2.577
	N-MCID vs. HC	1	Bilateral Anterior cingulate and paracingulate gyri Medial cingulate and paracingulate gyri	−2.584	NA		
		2	Right Insula Caudate nucleus Putamen	−2.578			
	MCID vs. N-MCID	NA			NA		
ROI 8	MCID vs. HC	1	Left Middle temporal gyrus Inferior temporal gyrus	4.207	1	Left Angular gyrus Middle temporal gyrus Middle occipital gyrus	4.495
		2	Right Precuneus Superior parietal gyrus Superior occipital gyrus Cuneate	−2.583	2	Right Superior temporal gyrus supramarginal gyrus Rolandic operculum Heschl’s gyrus Precentral gyrus	−2.576
		3	Right Precentral gyrus Postcentral gyrus Supramarginal gyrus	−2.576	3	Left Middle occipital gyrus Middle temporal gyrus Inferior occipital gyrus Inferior temporal gyrus	−2.581
		4	Right Superior parietal gyrus Postcentral gyrus Precuneus	−2.579			
		5	Right Caudate nucleus Thalamus	−2.582			
		6	Right Middle frontal gyrus Superior frontal gyrus	−2.579			
		7	Left Superior parietal gyrus Precuneus	−2.596			
	N-MCID vs. HC	1	Bilateral Precuneus	−2.578	1	Left Angular gyrus Middle temporal gyrus Middle occipital gyrus Inferior parietal gyrus	4.066
	MCID vs. N-MCID	NA			NA		

In the acupuncture state, the MCID group generally exhibited significantly lower FC between the feature ROIs and other brain regions compared to HC (e.g., ROI 1 with the bilateral cerebellum, left MOG, left MTG, and left ANG), but only a small proportion of higher FC between ROI 7 (i.e., the right PoCG, right PreCG, right rolandic operculum, and right IFG opercular part) with the right supramarginal gyrus (SMG), and right ANG; ROI 8 with the left ANG, left MTG and left MOG. There were two ROIs that exhibited stronger FC with other brain regions in the N-MCID group than in HC, mainly including ROI 5 with the left precuneus, bilateral LING, left cerebellum, left PCC, bilateral MFG orbital part, and ROI 8 with the left ANG, left MTG, left MOG, and left inferior parietal gyrus (IPG). There were no significant differences in FC between the other ROIs and the whole brain regions compared to HC. ROI 1, ROI 4, ROI 5, and ROI 6 presented lower FC with other brain regions in the MCID group than the N-MCID group. There were no significant differences in all FCs between the three groups of participants in the resting and acupuncture states, but patients exhibited fewer brain regions with abnormal FC in the acupuncture state than in the resting state.

## 4. Discussion

In this study, we applied a machine learning analysis method to screen functional brain regions that can classify clinical differences of motor recovery in patients with unilateral motor pathway injury (basal ganglia and/or corona radiata) after ischemic stroke. We identified eight regions with predicted weights>5% as regions of interest (ROIs) and found that these ROIs were located bilaterally in the cerebral hemispheres (Figure 3C). The weights of the contralesional brain regions accounted for approximately 35.16% (ROI 1, ROI 3, and ROI 4; Table 3) and the ipsilesional brain regions accounted for approximately 40.75% (ROI 2, and ROI 5–8; Table 3), suggesting that motor recovery after unilateral motor pathway injury is closely related to the regulation of bilateral brain regions. This was consistent with the point that the pattern of bilateral actions may contribute to engaging ipsilateral motor pathways in a motor behavior (Tazoe and Perez, 2014).

Motor rehabilitation can be affected by many factors. Because of this, we chose the MCID as a basis for our grouping, since it is the smallest improvement that patients can perceive as beneficial and focuses on the patient’s self-perception. The feature brain regions we obtained, mainly including the contralesional CAU (Graff-Radford et al., 2017), the bilateral SMA (Liu et al., 2022), PreCG (Park et al., 2011), PoCG (Ward et al., 2003), and PCL (Kang and Kim, 2008), were observed to be associated with motor behavior and outcomes in previous studies. There is bilateral interaction effect between the cerebral hemispheres. The balance between the bilateral hemispheres is disrupted after stroke and there is spontaneous functional regulation between the bilateral hemispheres to facilitate recovery. We found that the feature ROIs mainly involved the basal ganglia area (i.e., the location of the lesion corresponding to the contralesional hemisphere) and distal motor-related regions (e.g., SMA, PreCG, etc.) in the constralesional hemisphere, whereas mainly involved the distal motor-related regions in the ipsilesional hemisphere. This indicates that: (1) the distal regions of the ipsilesional hemisphere is important in predicting recovery after stroke, performed the intra-hemisphere adjustment; (2) the basal ganglia area of the contralesional hemisphere may compensate for the ipsilesional hemisphere, which may perform the compensate ability between two hemispheres; (3) functional synergistic changes in the distal motion-related regions of the bilateral hemispheres affected motor recovery through bilateral modulation, which may perform the interaction between two hemispheres.

Beside of these, we also found some brain regions with predictive weights related to mental and psychological aspects, such as the contralesional putamen (Klingbeil et al., 2022), the contralesional occipital lobe (Park et al., 2011), the ipsilesional frontal lobe (Stangeland et al., 2018), the ipsilesional orbital gyrus (Pan et al., 2022), etc. Some studies have also reported that psychosocial factors and non-motor brain regions have an impact on stroke rehabilitation (Ward et al., 2003; Qian et al., 2019). As can be seen, the ROIs we extracted involved motor and mental brain regions, and could be used as features to more comprehensively predict the classification of motor recovery after stroke.

Furthermore, we also extracted and compared the DC values of the ROIs, and found a statistically significant difference in DC values for only two ROIs (i.e., ROI 1, ROI 2) between the two groups. It is illustrated that machine learning can identify unexpected informative variables by traditional statistics and capture new potential features (Deo, 2015). Herein, the ML classifier achieved a balance accuracy of 75.51% and an AUC of 80.00%, indicating a relatively good performance. Therefore, we applied machine learning algorithms to classify patients with clinical differences in motor outcomes, identifying more personalized features of predicting motor outcomes in order to provide novel and referable neuroimaging evidence toward precision medicine for motor recovery after stroke (Deo, 2015; Handelman et al., 2018).

Subsequently, we compared the differences in FC among MCID, N-MCID, and HC in the resting and acupuncture states, respectively, to discover the brain functional effects of motor impairment and recovery after stroke. We demonstrated the response patterns of these characteristic ROIs in motor recovery and under acupuncture intervention and explored possible central neural mechanisms of acupuncture (Table 4; Figure 4; Supplementary Figure S1). In the resting state, compared to HC, abnormal FCs were found in patients and most of them exhibited lower FC, indicating that the synergy of different brain regions was reduced after stroke. Previous studies reported decreased functional connectivity between hemispheric brain regions in the early stages of stroke (Fan et al., 2015; Tang et al., 2016; Hensel et al., 2022) and concluded that hemispheric interactions in stroke patients were frequently characterized by abnormalities, in terms of balance and competition (Casula et al., 2021). Our results demonstrated that the abnormal form of FC exhibited was not entirely consistent between the different groups. In the MCID group, there were decreased FCs between the ipsilesional ROIs and the bilateral cerebral hemispheres, whereas the contralesional ROIs mainly presented decreased FCs between the ipsilesional hemisphere. In the N-MCID group, the decreased FCs were generally either between the ipsilateral or contralateral hemispheres of the ROIs. The lower FCs were found to be restored and can reach or even exceed the level of healthy controls (Park et al., 2011; Chen et al., 2020; Hensel et al., 2022) during motor recovery. Thus, the regulation and balance of interhemispheric inhibition can enhance post-stroke motor recovery (Boddington and Reynolds, 2017).

In the acupuncture state, we found that the MCID group still exhibited decreased FCs, but the range of brain regions with lower FC was reduced compared to the resting state. These results showed that acupuncture eliminated abnormal FCs between the contralesional ROIs with the ipsilesional brain regions, and between the ipsilesional ROIs within the ipsilesional brain regions. While in the N-MCID group, acupuncture restored the decreased FCs and even increased FCs between a part of the ipsilesional ROIs (ROI 5, ROI 8) with the contralesional brain regions. It could be speculated that acupuncture may have potential and specific targets of effects between different groups. Previous research considered that acupuncture can selectively adjust brain regions thought to be involved in mediating stroke recovery *via* functional plasticity (Li et al., 2006) and modulate functional connectivity between brain regions, brain networks, and hemispheres, which may be a beneficial effect of acupuncture to promote motor recovery from stroke (Qi et al., 2014; Li et al., 2015; Han et al., 2020). These findings above have similarities to our results, we suggested that acupuncture can modulate the bilateral hemispheres through feature ROIs to restore brain functional connectivity in stroke patients toward healthy controls and has its unique pattern of effects.

Notably, we noticed that the MCID group exhibited a wider range of brain regions with abnormal FCs than the N-MCID group, both in the resting and acupuncture states, although there was no statistically significant difference between the two groups. There are two possible reasons for this result, the first is that the MCID group has a higher class accuracy (82.14%) in machine learning. Therefore, the feature ROIs we extracted may be more representative for the MCID group. The second is that the basis of the grouping caused a more discrete distribution of the degree of motor impairment among the patients in the N-MCID group, most patients with limited recovery from severe impairments or littler recovery from mild impairments. It has been suggested that the outcome of brain function remodeling is related to the degree of initial damage. Different patterns of functional and structural reorganization of brain function exist in patients with different levels of deficits, leading to different prognoses. Changes in brain functional remodeling are often associated with clinical evaluation in patients with a mild degree of impairment and good recovery. In contrast, there is no correlation between patients with poor recovery and severe impairments (Lin et al., 2019; Jimenez-Marin et al., 2022). As a result, the N-MCID group may not express more differences.

There are several limitations to this study. The first is the follow-up time. We investigated the changes in FMA within 2 weeks of recovery from motor deficits. During the follow-up, there were some subjects drop out from the study, which could influence the accuracy of research. The period of recovery is 6 months after the stroke. Large-quantity and long-term longitudinal observations may uncover functional reorganization throughout the motor recovery period. Secondly, to avoid other redundant distractions, we chose a single acupoint for this study. However, this was not consistent with real clinical therapeutic protocols. In the future, studies about multiple acupoints can be carried out to reveal the mechanisms of acupuncture. The third limitation is the subgroup analysis. The focus of this study was on motor improvement in patients, but the recovery of motor function was related to the degree of initial motor impairment. Further subgroup analysis of the degree of initial impairment could provide a deeper understanding of stroke rehabilitation.

## 5. Conclusion

In this study, we applied a machine learning approach to identify the feature ROIs that can predict the classification of the MCID for motor improvement after ischemic stroke, and then compared the brain functional connectivity and acupuncture effects of these brain regions. Motor impairment and recovery result from the co-regulation of the bilateral cerebral hemispheres, and different brain functional response patterns exist in patients with different motor outcomes. Acupuncture can modulate the bilateral hemispheres through feature ROIs and eliminate abnormal functional connectivity to promote motor recovery after ischemic stroke. Our study can provide potential neuroimaging features for motor recovery and mechanisms of acupuncture on functional organization after stroke, and may expand research thoughts of machine learning and fMRI in clinical applications.

## Data availability statement

The original contributions presented in the study are included in the article/Supplementary material, further inquiries can be directed to the corresponding authors.

## Ethics statement

The studies involving human participants were reviewed and approved by Dongzhimen Hospital Affiliated to Beijing University of Chinese Medicine Institutional Review Boards. The patients/participants provided their written informed consent to participate in this study.

## Author contributions

ML and ZD were involved in literature search, data analyses and writing of the manuscript. JZ contributed to the experimental design. RL and LJ were involved in data analyses and reviewing the manuscript. LX and XY contributed to the clinical observations. MZ and TX contributed to the subjects’ recruitment. JW and WW were involved in clinical diagnosis for stroke patients. CC contributed to FMA evaluations. JF designed the imaging approaches and consulted through the study. YZ designed the study protocol and sought funding. All authors read and approved the final manuscript.

## Funding

This work was supported by the National Natural Science Foundation of China (grant numbers 81873257, 81473667, and 82174331).

## Conflict of interest

The authors declare that the research was conducted in the absence of any commercial or financial relationships that could be construed as a potential conflict of interest.

## Publisher’s note

All claims expressed in this article are solely those of the authors and do not necessarily represent those of their affiliated organizations, or those of the publisher, the editors and the reviewers. Any product that may be evaluated in this article, or claim that may be made by its manufacturer, is not guaranteed or endorsed by the publisher.

## Footnotes

^1^ http://rfmri.org/DPABI

^2^ http://www.fil.ion.ucl.ac.uk/spm

^3^ http://www.mlnl.cs.ucl.ac.uk/pronto/
