# Supplementary material for: Neuroimaging mechanisms of acupuncture on functional reorganization for post-stroke motor improvement: a machine learning-based functional magnetic resonance imaging study
Source: Front Neurosci. 2023 May 19;17:1143239. doi: 10.3389/fnins.2023.1143239 (PMC10235506; doi:10.3389/fnins.2023.1143239)
Supplement: Supplementary file 1 [file Data_Sheet_1.PDF]

Supplement Figure 1. Plots of FC differences between feature ROI 3 - 8 with the whole brain in the resting and acupuncture states. The blue clusters in the former group indicate lower FC between these brain regions with the ROI, while the red clusters indicate higher FC between these brain regions with the ROI, compared to the latter group. Figure a-1, b-1, c-1, d-1, e-1, and f-1 present the anatomical position and weights distribution of feature ROI 3 - 8. Red indicates high weight, and blue indicates low weight. FC, Functional Connectivity; ROI, Regions of Interest; MCID, Minimal Clinically Important Difference; N-MCID, Non-Minimal Clinically Important Difference

a-1 ROI 3

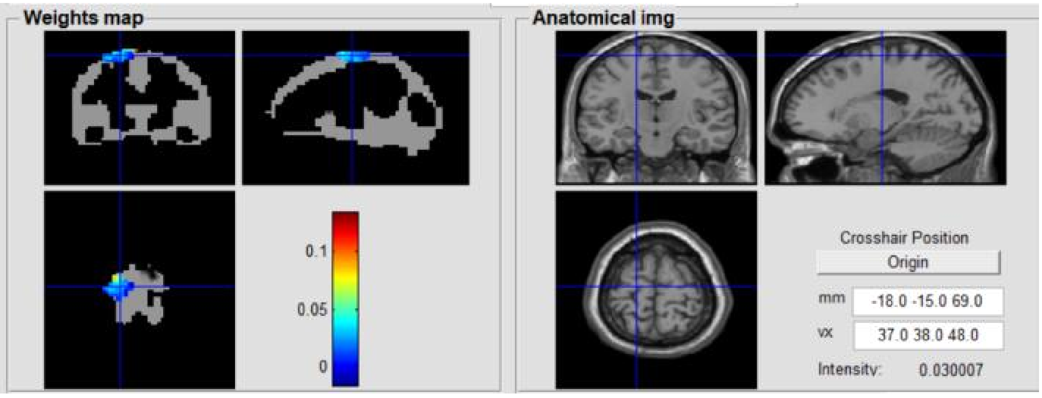

a-2

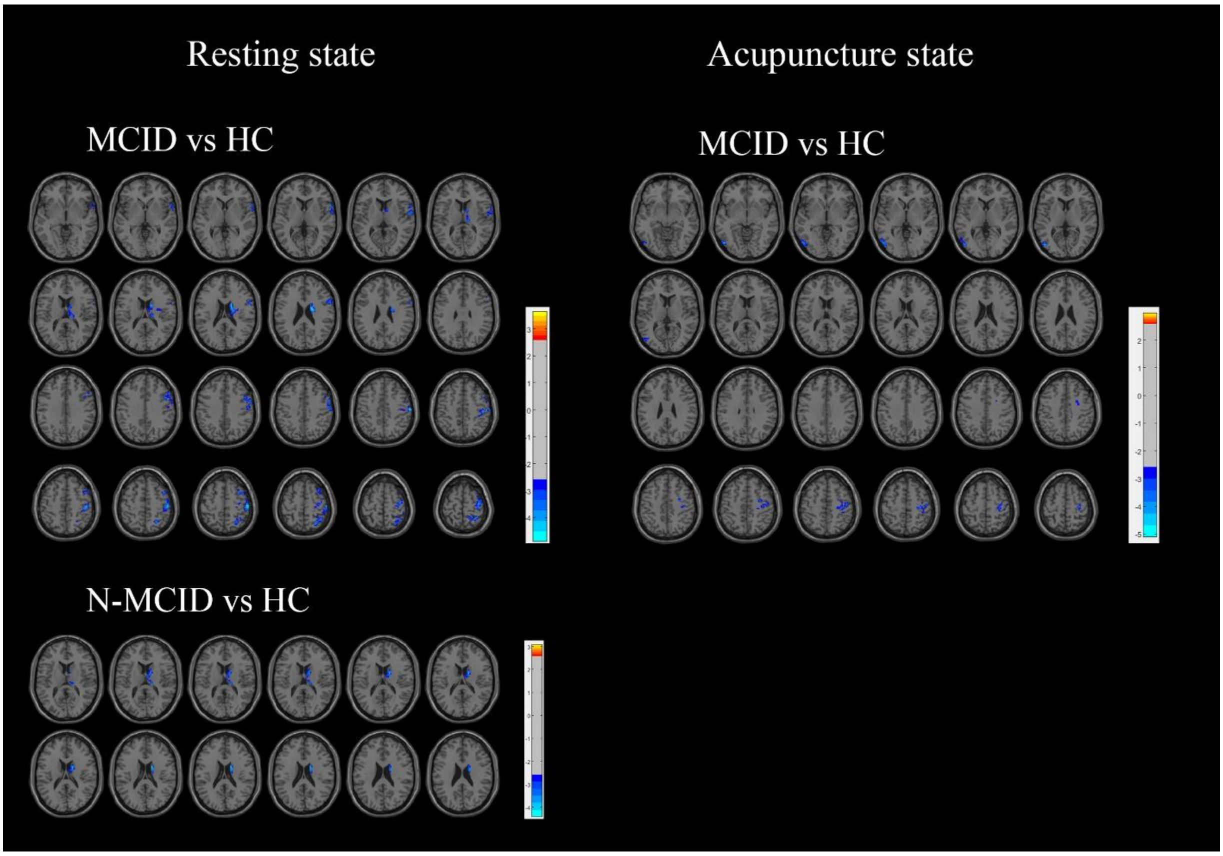

b-1 ROI 4

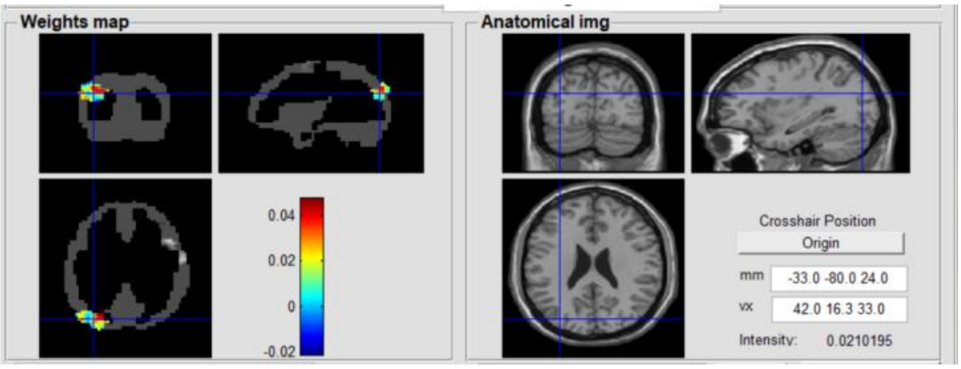

b-2

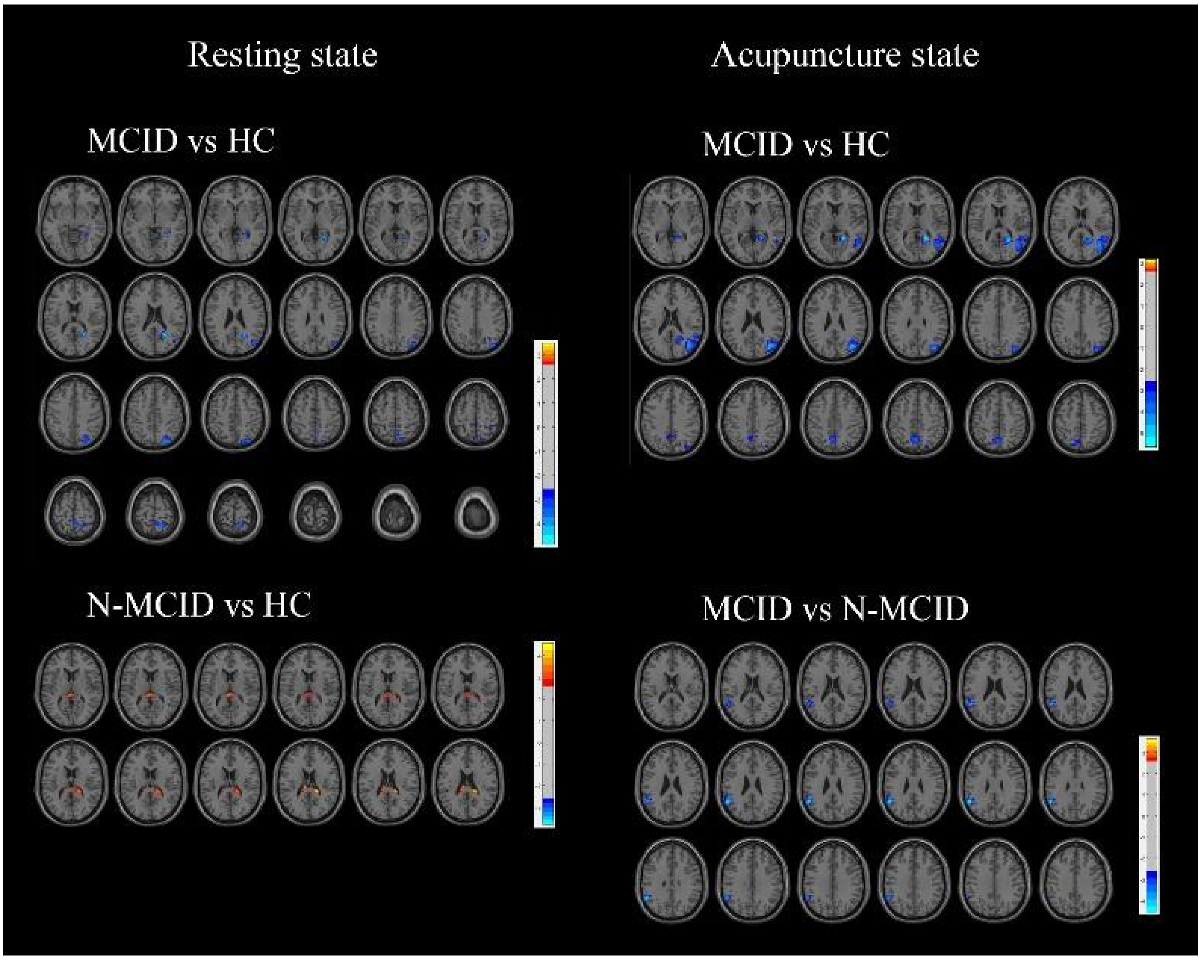

c-1 ROI 5

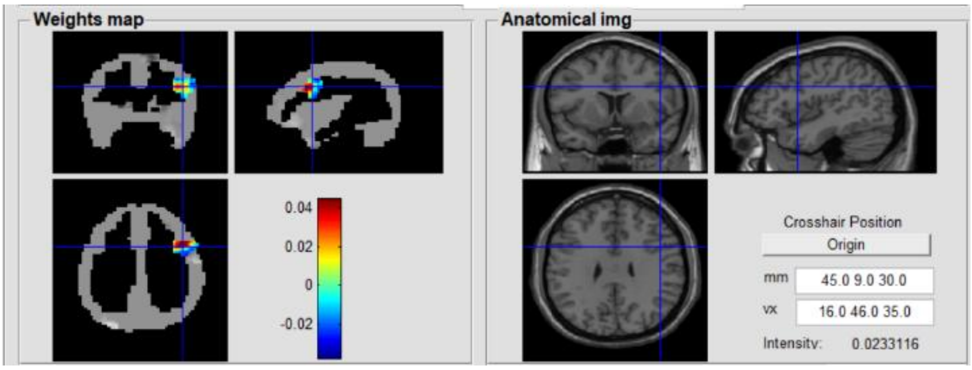
Weights map
Anatomical img
0.04
0.02
0
-0.02
Crosshair Position
Origin
mm
45.0 9.0 30.0
vx
16.0 46.0 35.0
Intensity:
0.0233116

c-2

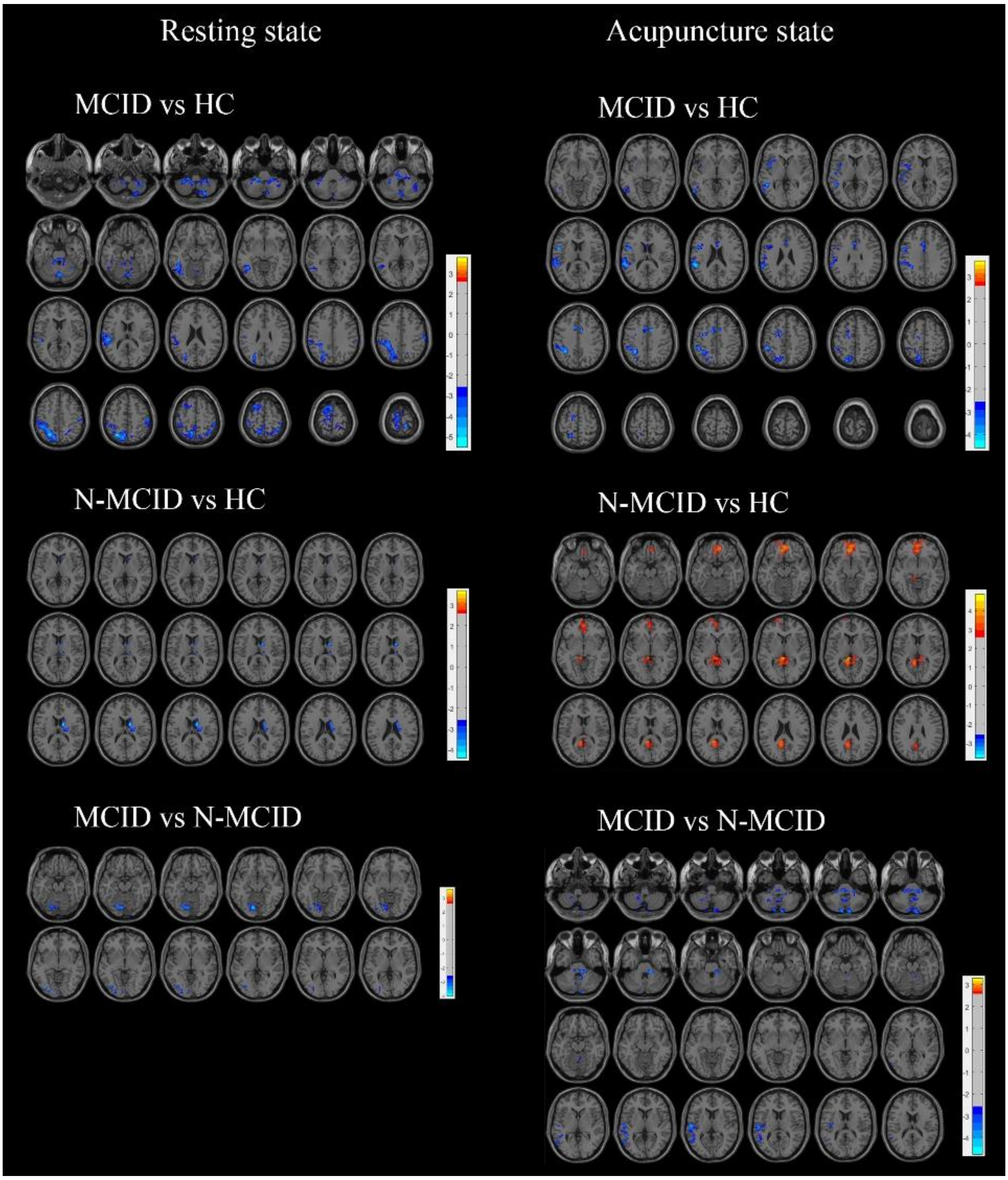
Resting state
Acupuncture state
MCID vs HC
MCID vs HC
N-MCID vs HC
N-MCID vs HC
MCID vs N-MCID
MCID vs N-MCID

d-1 ROI 6

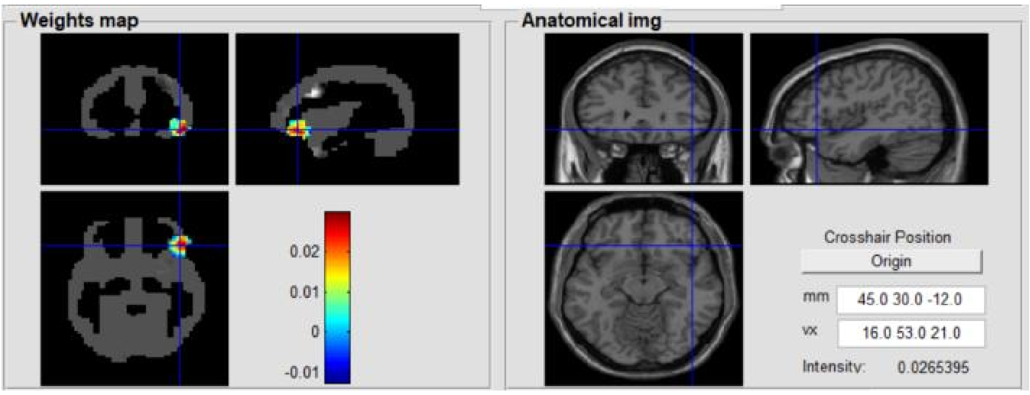
Weights map
0.02
0.01
0
-0.01
Anatomical img
Crosshair Position
Origin
mm 45.0 30.0 -12.0
vx 16.0 53.0 21.0
Intensity: 0.0265395

d-2

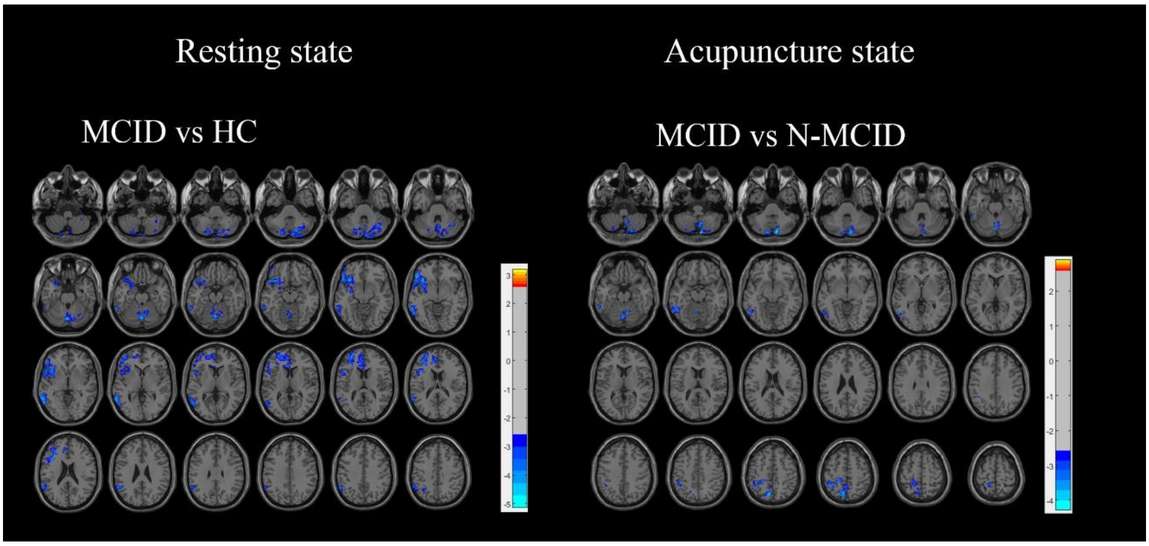
Resting state
Acupuncture state
MCID vs HC
MCID vs N-MCID

e-1 ROI 7

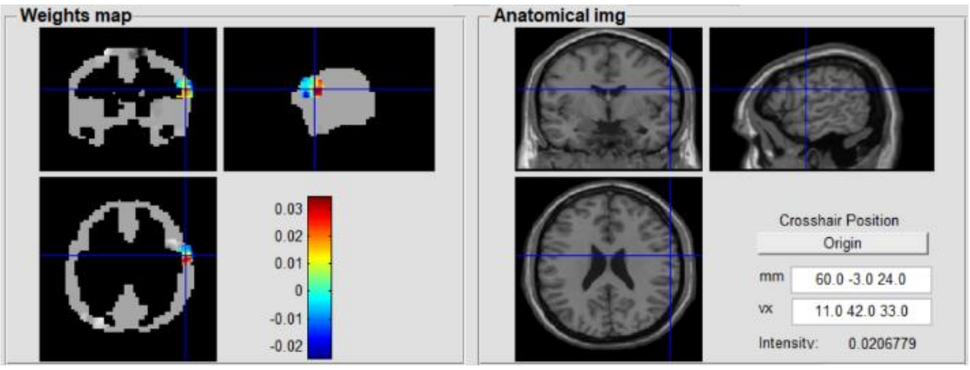
Weights map
0.03
0.02
0.01
0
-0.01
-0.02
Anatomical img
Crosshair Position
Origin
mm 60.0 -3.0 24.0
vx 11.0 42.0 33.0
Intensity: 0.0206779

e-2

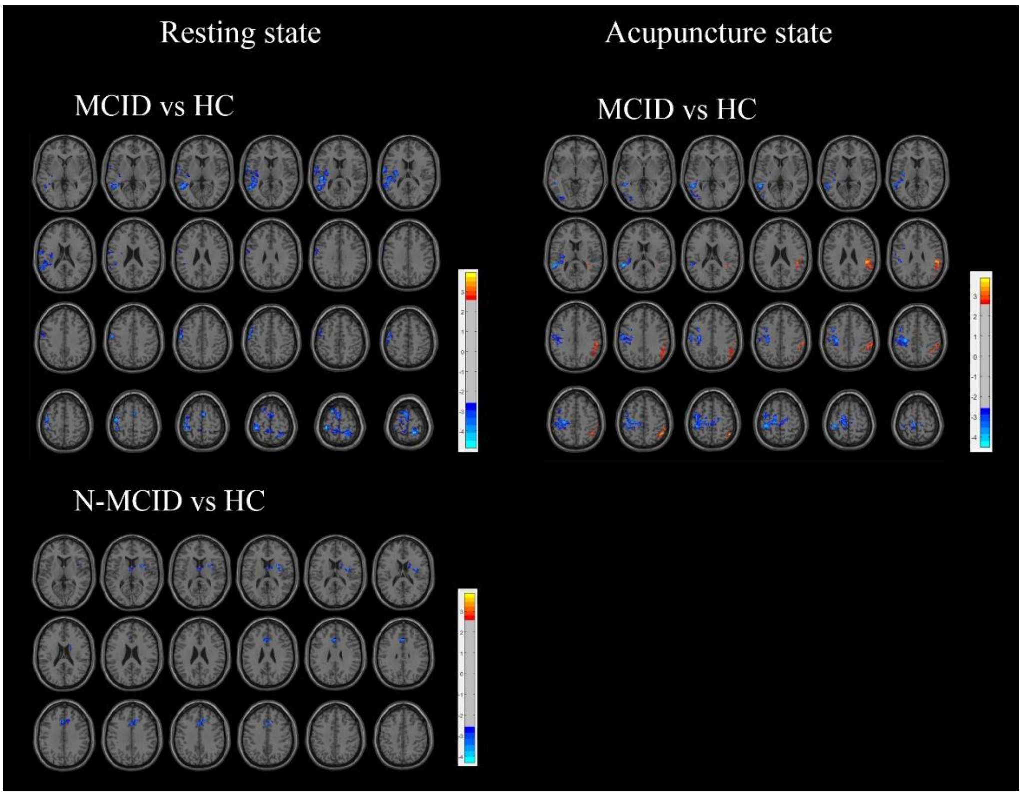
Resting state
Acupuncture state
MCID vs HC
MCID vs HC
N-MCID vs HC

f-1 ROI 8

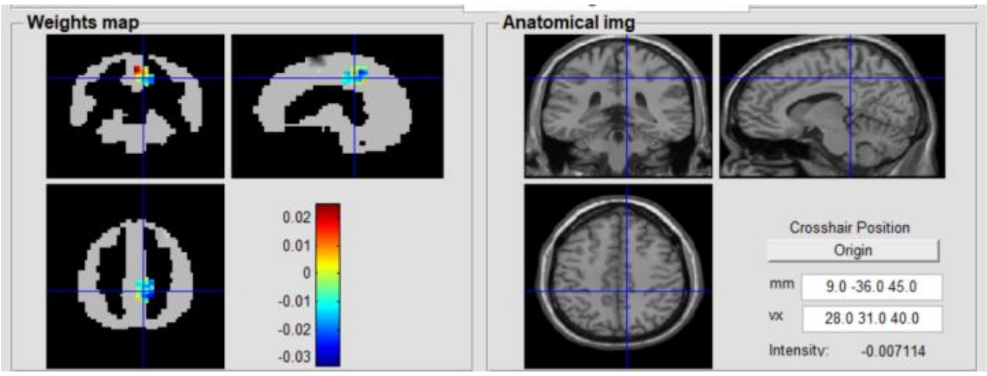
Weights map
Anatomical img
0.02
0.01
0
-0.01
-0.02
-0.03
Crosshair Position
Origin
mm
9.0 -36.0 45.0
vx
28.0 31.0 40.0
Intensity:
-0.007114

f-2

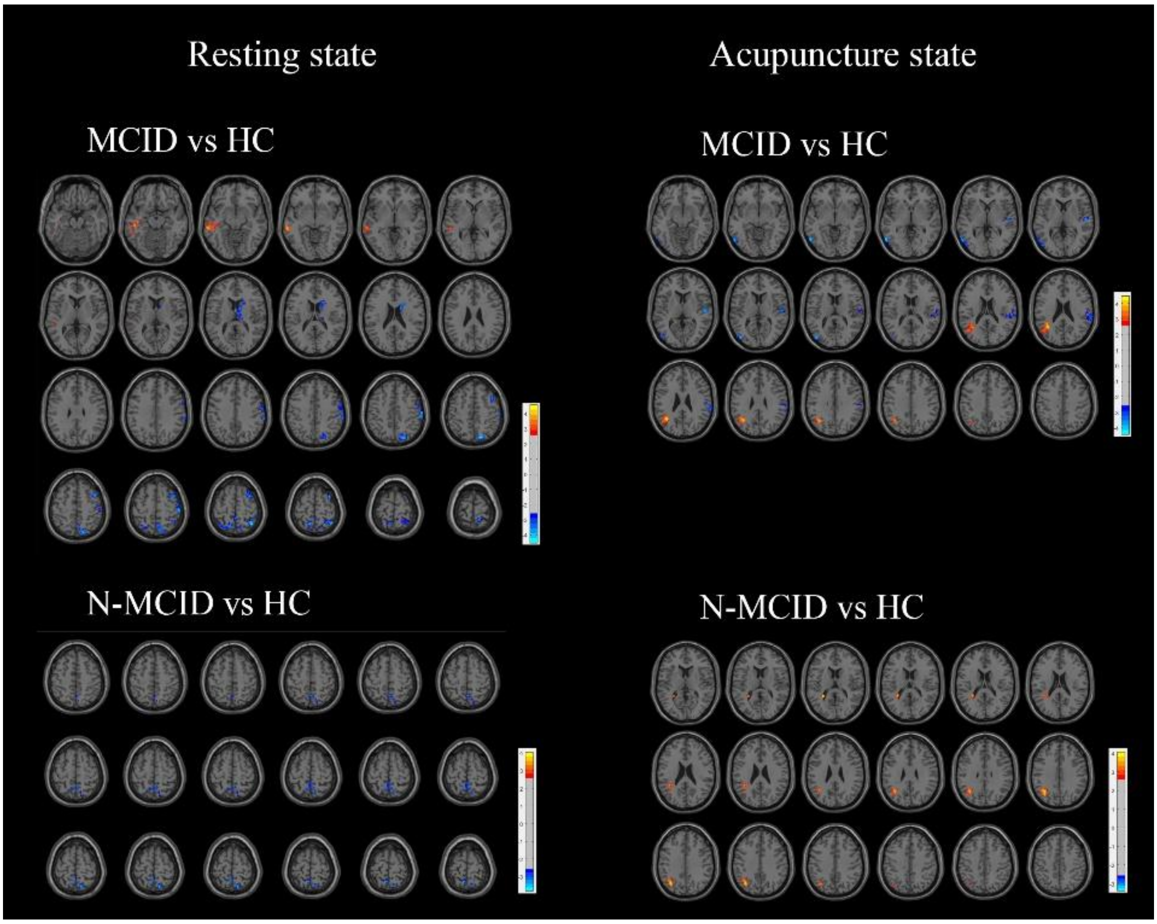
Resting state
Acupuncture state
MCID vs HC
MCID vs HC
N-MCID vs HC
N-MCID vs HC

Supplement Table 1. Details of groups.

Group	Sex	Age	Course of Disease (days)	Lesion side	Location of lesion	Baseline			Follow-up		
						FMA-UM	FMA-LE	FMA-TOTAL	FMA-UM	FMA-LE	FMA-TOTAL
**MCID**											
1	Male	43	11	left	lateral ventricular, basal ganglia	5	13	18	7	27	34
2	Male	62	6	left	lateral ventricular, basal ganglia	64	16	80	66	30	96
3	Male	65	9	right	basal ganglia	57	29	86	62	32	94
4	Female	68	26	left	basal ganglia, corona radiata	36	34	70	41	34	75
5	Female	66	53	right	basal ganglia, corona radiata	11	9	20	16	15	31
6	Male	71	5	right	basal ganglia	55	30	85	61	31	92
7	Male	60	35	left	basal ganglia	14	13	27	20	23	43
8	Female	62	18	right	lateral ventricular	17	31	48	26	32	58
9	Female	57	35	right	lateral ventricular	54	21	75	63	31	94
10	Male	52	5	left	lateral ventricular, basal ganglia	51	28	79	61	33	94
11	Male	68	24	right	lateral ventricular	56	28	84	66	29	95
12	Female	65	20	right	lateral ventricular, corona radiata	53	34	87	63	34	97
13	Female	62	19	left	lateral ventricular, basal ganglia	48	32	80	58	32	90
14	Female	53	31	right	lateral ventricular	6	28	34	17	29	46
15	Female	61	20	right	basal ganglia	42	34	76	53	34	87
16	Male	32	13	left	basal ganglia	38	31	69	49	32	81
17	Male	63	41	right	basal ganglia	12	16	28	24	20	44
18	Male	61	18	left	basal ganglia, corona radiata	38	29	67	51	34	85
19	Male	48	30	right	frontal lobe, parietal lobe, occipital lobe, lateral ventricular, basal ganglia	19	31	50	34	32	66
20	Male	63	11	right	basal ganglia	44	4	48	60	18	78

21	Male	55	8	left	lateral ventricular, basal ganglia	35	21	56	52	30	82
22	Male	72	30	left	lateral ventricular, basal ganglia	23	25	48	43	28	71
23	Male	67	9	left	basal ganglia	6	6	12	29	15	44
24	Female	69	11	left	lateral ventricular, basal ganglia	21	24	45	46	30	76
25	Female	73	15	right	lateral ventricular, basal ganglia	26	34	60	53	33	86
26	Male	57	5	left	basal ganglia	0	34	34	31	34	65
27	Female	71	58	right	basal ganglia, corona radiata	9	11	20	10	17	27
28	Male	60	69	right	basal ganglia, corona radiata	15	24	39	21	28	49
**N-MCID**											
29	Male	47	3	right	lateral ventricular	61	30	91	51	32	83
30	Male	62	15	right	frontal lobe, parietal lobe, basal ganglia near caudate nucleus	62	33	95	57	31	88
31	Male	72	21	right	lateral ventricular, basal ganglia, ocipital lobe	16	14	30	14	12	28
32	Male	62	3	right	lateral ventricular, basal ganglia	10	13	23	9	15	24
33	Female	70	13	right	lateral ventricular, basal ganglia	59	29	88	58	30	88
34	Female	61	14	left	lateral ventricular, basal ganglia	4	4	8	4	9	13
35	Male	50	21	right	basal ganglia, corona radiata	5	2	7	5	7	12
36	Male	77	31	right	lateral ventricular, centrum semiovale	57	32	89	58	32	90
37	Male	66	27	left	basal ganglia	4	22	26	5	25	30
38	Male	60	48	right	centrum semiovale, corona radiata, occipitoparietal areas	6	15	21	7	15	22
39	Male	68	6	left	lateral ventricular, basal ganglia	57	32	89	59	32	91
40	Male	65	4	left	centrum semiovale	62	34	96	64	34	98
41	Male	49	7	right	basal ganglia	12	18	30	14	18	32
42	Male	35	6	left	basal ganglia	7	34	41	9	34	43
43	Female	71	12	right	basal ganglia	0	15	15	2	19	21

44	Female	65	31	right	basal ganglia	57	26	83	60	27	87
45	Male	57	57	right	periventricular	7	21	28	11	20	31
46	Male	75	22	right	basal ganglia, corona radiata	7	24	31	11	27	38
47	Male	65	42	right	lateral ventricular	55	33	88	59	33	92
48	Female	42	37	left	lateral ventricular, basal ganglia	35	20	55	39	26	65
49	Female	58	24	right	lateral ventricular, basal ganglia	12	18	30	16	22	38
**HC**											
50	Male	73									
51	Female	68									
52	Male	68									
53	Male	65									
54	Male	63									
55	Female	63									
56	Male	62									
57	Female	62									
58	Female	62									
59	Female	62									
60	Male	62									
61	Male	61									
62	Female	60									
63	Male	59									
64	Male	59									
65	Female	59									
66	Female	55									
67	Male	54									
68	Male	52									
69	Male	53									
70	Male	51									
71	Male	48									
72	Female	57									

73	Male	40
74	Female	56
75	Male	53

Abbreviations: MCID, Minimal Clinically Important Difference; N-MCID, Non-Minimal Clinically Important Difference; HC, Healthy Controls; FMA-UE, Fugl-Meyer Assessment Upper Extremity scores; FMA-LE, Fugl-Meyer Assessment Lower Extremity scores; FMA-Total, Fugl-Meyer Assessment Total scores.
